# Annotations

**Published:** 1911-01-28

**Authors:** 


### Annotations.

### Sir Francis Galton.

Like his even more renowned cousin, Charles Darwin, the late Sir Francis Galton, who died last week at the age of eighty-eight, was one of that leisured and moneyed class to whom the term of idle rich is applied in the current demagogy of the day. Whatever justification this phrase may have in many cases, the careers of these two cousins afford scant target for the arrows of abuse; for of two men more abundantly occupied throughout life it would be hard offhand to give the names. The mere recital of the many branches of study in which Galton interested himself after completing his medical education almost takes away one's breath; and when it is reflected that this was no superficial student, but an original thinker, who founded one new science and profoundly modified others, the estimation of his genius is bound to develop into respect and admiration. Almost sixty years ago the deceased scientist's travels in the Sudan, Bechuanaland, Damaraland, and other parts of Africa earned him the gold medal of the Royal Geographical Society. This work, be it noted, makes Sir Francis Galton the predecessor of Stanley, Cameron, Livingstone, Thomson, and almost all the other great African explorers of the Victorian age—themselves now hardly more than memories to a generation which is already past its prime. Since those early successes the tale of achievement is a long one. The entire reformation and rehabilitation of meteorology alone would constitute a reasonable title to fame for most men, but it was but one among Galton's many activities. It is, however, quite probable that when all these are forgotten, Galton will still be remembered as the author of the modern conception of eugenics. Whether he borrowed his idea from Plato or any one else, he impressed his own individuality upon the subject so strongly that he must always be regarded as the founder of this new science, or, as it may better be termed, this new school. What its future is to be is yet to be determined; it may be that Galton has here been a century or two ahead of his times. But when the nineteenth and twentieth centuries come to be reviewed from the perspective of the twenty-first and twenty-second there will surely be few figures more esteemed and revered than that of the subject of this memoir.

### The Education and Examination of Pharmacists.

It is not unlikely that in the near future the system of pharmaceutical education and examination now in force in this country will undergo a complete change. Under the existing system candidates for the qualifying examination in pharmacy are at liberty to acquire their knowledge when and where they may, and, as a consequence, an exceptionally large proportion of them are not properly trained, and fail to satisfy the examiners. The Council of the Pharmaceutical Society, which is the statutory examining authority, from 1887 onwards, made repeated attempts to obtain legislation to remedy the defects of the system, and in 1908 succeeded in obtaining powers to institute a compulsory curriculum and to divide the examination into two parts. The Council proposes to put these powers into effect, and as a first step has submitted a draft scheme to schools and colleges of pharmacy and to local organisations for their consideration. This scheme does not suggest any change in the examination syllabus, but proposes to divide the examination into two parts and to lay down certain conditions to be fulfilled by candidates. It is proposed to require candidates entering for the first examination to produce evidence of having passed the preliminary examination and of having attended fifty lectures in botany, a hundred lectures in chemistry, twenty-five lectures in physics, and of having done twenty-five hours' work in practical botany and three hundred hours' work in practical chemistry at a teaching institution approved by the Council. Having passed the first part, candidates are eligible to enter for the second part, provided they have been engaged for three years after registration as students in the ordinary work of pharmacy under the supervision of a registered pharmacist; they will also be required to produce evidence of practical work. Should the proposed scheme be put into effect the percentage of unsuccessful candidates would without doubt be sensibly diminished, and a highly-trained class of men would be provided for the practice of pharmacy.

### A Nice Point.

The intricate problems of medico-legal interest which the various Workmen's Compensation Acts have occasioned seem to be never-ending. As recently as January 9 a curious case of this sort came before the City of London Court. It was that of a navvy who sustained an inguinal hernia whilst at work, and was paid half-wages for three months under a ruling which now seems to be generally accepted in these cases. The employers then refused to pay any more on the ground that a radical cure would restore him to efficiency. The man went to St. Bartholomew's Hospital and was admitted; but after having him under observation for eight days the surgeon decided not to operate, as it was considered that he was a bad subject for an anæsthetic owing to alcoholism, and the man was accordingly discharged. The question of spinal anæsthesia does not seem to have been considered; at least, no mention of it is made in the report, yet it may be well argued that such a case is pre-eminently that for which spinal anæsthesia is indicated. On the other hand, evidence was given for the defence that there was no undue risk to be apprehended; and the case was settled owing to the patient's willingness to go elsewhere for the operation. Thus the exact law of such a situation was not laid down, as by consent the employers agreed to pay the compensation up to date, and the man agreed to have a radical cure. But if the latter had refused this, it would be difficult for any tribunal to terminate the award in face of the evidence from St. Bartholomew's.

